# Beyond Cartilage Repair: The Role of the Osteochondral Unit in Joint Health and Disease

**DOI:** 10.1089/ten.teb.2018.0122

**Published:** 2019-04-16

**Authors:** Sarah I.M. Lepage, Naomi Robson, Hillary Gilmore, Ola Davis, Allyssa Hooper, Stephanie St. John, Vashine Kamesan, Paul Gelis, Diana Carvajal, Mark Hurtig, Thomas G. Koch

**Affiliations:** ^1^ Department of Biomedical Sciences, University of Guelph, Guelph, Canada.; ^2^ Department of Clinical Studies, University of Guelph, Guelph, Canada.

**Keywords:** cartilage, bone, osteochondral unit, crosstalk, tissue engineering

## Abstract

Once believed to be limited to articular cartilage, osteoarthritis is now considered to be an organ disease of the “whole joint.” Damage to the articular surface can lead to, be caused by, or occur in parallel with, damage to other tissues in the joint. The relationship between cartilage and the underlying subchondral bone has particular importance when assessing joint health and determining treatment strategies. The articular cartilage is anchored to the subchondral bone through an interface of calcified cartilage, which as a whole makes up the osteochondral unit. This unit functions primarily by transferring load-bearing weight over the joint to allow for normal joint articulation and movement. Unfortunately, irreversible damage and degeneration of the osteochondral unit can severely limit joint function. Our understanding of joint pain, the primary complaint of patients, is poorly understood and past efforts toward structural cartilage restoration have often not been associated with a reduction in pain. Continued research focusing on the contribution of subchondral bone and restoration of the entire osteochondral unit are therefore needed, with the hope that this will lead to curative, and not merely palliative, treatment options. The purpose of this narrative review is to investigate the role of the osteochondral unit in joint health and disease. Topics of discussion include the crosstalk between cartilage and bone, the efficacy of diagnostic procedures, the origins of joint pain, current and emerging treatment paradigms, and suitable preclinical animal models for safety and efficacy assessment of novel osteochondral therapies. The goal of the review is to facilitate an appreciation of the important role played by the subchondral bone in joint pain and why the osteochondral unit as a whole should be considered in many cases of joint restoration strategies.

**Impact Statement:**

In this comprehensive review, we are providing a holistic overview of osteochondral tissue development, disease, pain localization, as well as structural evaluation and current repair strategies. This review is intended to serve as a broad introduction to this multidisciplinary research area. It is a thorough examination of the biological aspects of the osteochondral unit from a tissue engineering perspective, highlighting the importance of the subchondral bone in chondral and osteochondral lesion repair and pain relief.

## Introduction

The homeostasis between joint tissues, particularly between cartilage and the underlying subchondral bone, is fundamentally important to understand when determining treatment strategies for chondral, osteochondral, and subchondral bone lesions. If left untreated, these lesions can progress to osteoarthritis (OA), as the damage advances to widespread tissue degeneration along with severe joint pain and stiffness.^1^

Previous treatment strategies have had a strong focus on structural repair of the articular cartilage only, but in many cases, bone lesions cause the associated pain, and not articular cartilage damage.^2^ As nociceptors are present within the subchondral bone and not the cartilage,^3^ it would therefore seem prudent to consider the cartilage and subchondral bone as one entity to be successful in ameliorating joint pain while restoring the load-bearing and frictionless movement capacity of the healthy joint.

Recently improved imaging techniques have contributed to our understanding of the importance of bone pathologies and their contribution to OA progression and pain. For instance, posttraumatic bone bruising or bone marrow lesions (which can be visualized with magnetic resonance imaging [MRI]) can cause sustained bone remodeling that can lead to a loss of subchondral bone support for the cartilage. Moderate-to-severe bone marrow lesions that do not resolve over time are linked to ongoing symptoms and future cartilage degradation.^4–6^

In this review, we will focus on literature describing the crosstalk between cartilage, calcified cartilage and bone tissues, their response to injury, repertoire of repair responses, and their relationship to pain. This enables consideration of current and emerging treatment modalities for osteochondral defects as well as new tissue engineering tactics and animal models. We will link these broad topics in an effort to shed light on the limitations of today's treatment techniques and propose directions of future research to address the complexities of the development, regeneration, and repair of the osteochondral unit.

## Methods

A variety of databases were used to collect and review all referenced material, including University of Guelph Primo, PubMed through NCBI, Medline, ProQuest Biological Sciences, the Cochrane Database, and Google Scholar. The keywords used in the search of the databases were cartilage, bone, osteochondral unit, endochondral ossification, osteochondral crosstalk, interzone, joint biomechanics, joint pain, joint nociceptors, evaluation, MRI, arthroscopy, radiography, clinical trials, microfracture, autologous chondrocyte implantation (ACI), BioCartilage, particulate cartilage, cartilage autologous implantation system (CAIS), dual-tissue transplantation, osteochondral autograft transfer (OAT), mosaicplasty, tissue engineering, animal models, synovium, T2 mapping, and chondrocytes. References listed were identified through primary searches, articles known to the authors, or from reference lists from other review articles.

### Synovial joints and the osteochondral unit

Synovial joints are complex structures that permit near frictionless motion between bones to facilitate human ambulation. Hyaline cartilage, lining the bone ends in these joints, has the unique ability to withstand high loads and while maintaining a near-frictionless and compliant articulating interface between the bones. Load dispersal is shared with the subchondral bone plate and it is believed that the morphology of the subchondral bone is a direct expression and adaptation of past loading history in adults.^7^

**FIG. 1.**
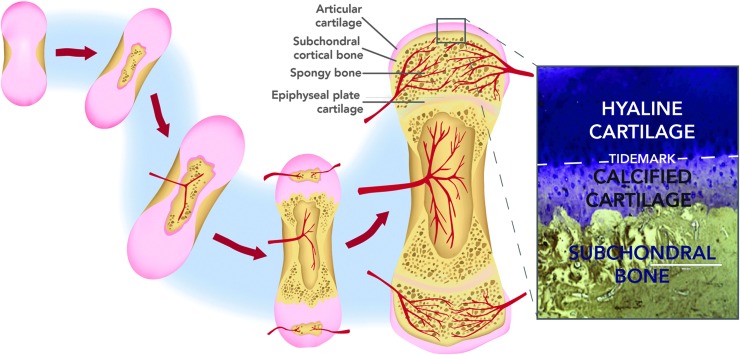
Diagram of endochondral ossification and the formation of the osteochondral unit. *Inset*: Histological view of the osteochondral unit (stained with Toluidine Blue and von Kossa).

This dynamic relationship between cartilage and bone (the osteochondral unit) is crucial to maintaining overall joint health and integrity. The osteochondral unit is composed of hyaline cartilage connected through a zone of calcified cartilage to the subchondral cortical bone known as the subchondral plate, which gives way to metaphyseal trabecular bone. The distinct histological boundary between hyaline and calcified cartilage is known as the tidemark (Fig. 1, inset).

The osteochondral unit arises as the final product of endochondral ossification, where the fetal cartilage “anlagen” is diminished by endochondral ossification after birth, leaving permanent articular cartilage at the ends of fully developed long bones. Upon initiation of ossification in the cartilage anlagen, bone tissue development occurs first in the diaphysis (primary ossification center) and then in the epiphyses (secondary ossification centers). Differentiating chondrocytes undergo hypertrophy and express collagen type X, which aids in promoting calcification of the surrounding matrix.^8,9^ Blood vessels invade the developing tissue, infiltrating it with osteoblasts and driving the expression of osteogenic factors such as Runx2, eventually replacing the transient cartilage with bone.^10,11^ A thin layer of calcified cartilage matrix remains at the distal ends of the long bones after ossification, anchoring the newly developed bone to the stable articular cartilage (Fig. 1).

Stable articular cartilage arises from the interzone in the developing limb, which separates epiphyseal ossification centers and serves as the eventual location of the joint. As chondrification proceeds toward the ends of the developing long bone, the interzone arises from the remaining condensed mesenchymal cells, forming a tightly packed cellular region between the ossification centers.^12^ Cells in this region form three distinct layers: two outer chondrogenic layers that become the lining of the epiphyses of long bones, and a middle layer that undergoes cavitation.^13^ It is speculated that the two outer layers mark the transition zone between calcified cartilage and the newly formed subchondral bone, while the middle layer forms the mature articular cartilage.^14^ The resulting articular cartilage and underlying subchondral bone form the components of the mature osteochondral unit.

Damage to or dysregulation of the osteochondral unit is often traumatic in nature, resulting in cartilage and/or subchondral bone lesions that, if unable to heal, most frequently results in OA, possibly the most chronic, debilitating disease among adults worldwide.^15^ Osteochondral defects typically arise in adults as a result of acute trauma to the cartilage and underlying bone or in association with meniscal/ligament tears. In young, active children and adults, osteochondral defects may form as a result of osteochondritis dissecans (OCD), a painful condition characterized by bone sclerosis that can lead to cartilage fragmentation. OCD likely develops through improper development, repetitive trauma, inflammation, and/or a decrease in blood supply, rather than an acute osteochondral fracture.^16^

Whatever the cause, focal damage to the osteochondral unit initiates a cascade of repair and remodeling attempts that often have detrimental effects on the long-term health and function of the joint that can lead to OA.^17^

### Crosstalk within the osteochondral unit

The onset of disease or degeneration of one component of the osteochondral unit can impact the functions of other components. Biomechanically, subchondral bone normally supports the articular cartilage in distributing joint forces over the joint. However, damage to the subchondral bone can alter its elastic modulus and thus its force distribution properties, which can lead to cartilage degeneration through abnormal loading on the tissue.^18,19^ Conversely, damage to the cartilage articular surface may occur before or concurrent with bone changes and remodeling.^20^ This biomechanical relationship is a well-established paradigm for OA progression; however, several studies have also evaluated the concept of a biochemical crosstalk between cartilage and bone tissues.

The proximity of the subchondral bone vasculature suggests that small molecules can perfuse into the cartilage in healthy osteochondral tissue. Key studies performed by Arkill and Winlove and Pan *et al.* demonstrated the transportation of fluorescent dyes from the subchondral circulation to deep zone cartilage.^21,22^ This diffusion has been shown to be elevated in OA.^23^ In addition, transport of larger molecules may occur through the osteocyte canalicular/lacunar network,^24^ which may be affected by the progression of OA with increasing subchondral bone porosity.^25^

**FIG. 2.**
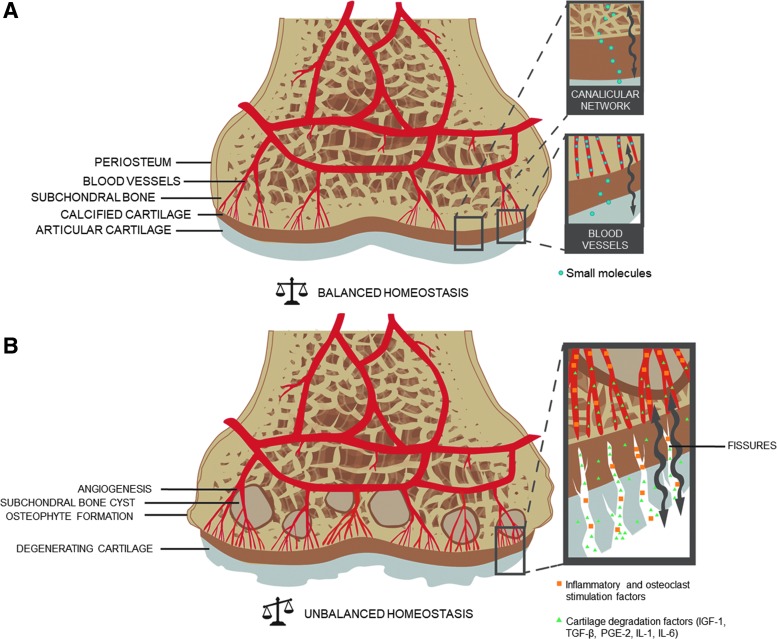
Proposed mechanism of crosstalk within the osteochondral unit. Small molecules can diffuse through blood vessels and the canalicular network in healthy synovial joints **(A)**. In osteoarthritic joints, in addition to the formation of osteophytes, subchondral bone cysts, and cartilage degeneration, angiogenesis and the formation of fissures may cause an increase in transport of inflammatory, osteoclast, and cartilage degradation factors **(B)**.

OA progression can also introduce fissures, microcracks, and new blood vessels that penetrate the calcified cartilage and may increase exchange of signaling molecules between the cartilage and bone.^26^ It has been suggested that cytokines and prostaglandins involved in bone tissue remodeling can reach the overlying cartilage through these new channels, further resulting in its catabolism. Conversely, inflammatory and osteoclast stimulation factors released by the synovial membrane and/or the articular cartilage could affect the subchondral bone^27^ (Fig. 2). This crosstalk between bone and cartilage may be partially responsible for the etiology and progression of OA, and is an important consideration when exploring new treatment modalities to heal osteochondral defects.

### Pain and the osteochondral unit

The relationship between cartilage and bone in the context of osteochondral unit degeneration is very important when assessing the cause of joint pain, the primary symptom of osteochondral lesions, and OA. As healthy hyaline cartilage does not contain nociceptors (pain receptors),^3^ joint pain originates from the underlying subchondral bone or other soft tissue components of the joint capsule, such as the synovium. Synovitis and its associated pain can occur concurrently with OA and subchondral bone lesions,^28–30^ but this is thought to be distinct from processes that regulate subchondral bone pain.^31^

In addition, subchondral bone lesions have been shown to be more highly correlated with pain than synovitis or joint effusion.^32,33^ Subchondral bone angiogenesis during early OA progression may facilitate not only the increased crosstalk leading to cartilage degradation, but also the innervation of the overlying cartilage.^34^ Walsh *et al.* examined the link between new microvasculature and nerve growth in osteochondral tissue and found increased vascular endothelial growth factor (VEGF) and nerve growth factor (NGF) in the subchondral bone space, breaching into the noncalcified cartilage.^31^ They postulated that the increase in VEGF (a proangiogenesis factor) contributed to the growth of new blood vessels in articular cartilage, which was accompanied by nociceptor growth through NGF (a neurotrophic factor).

**FIG. 3.**
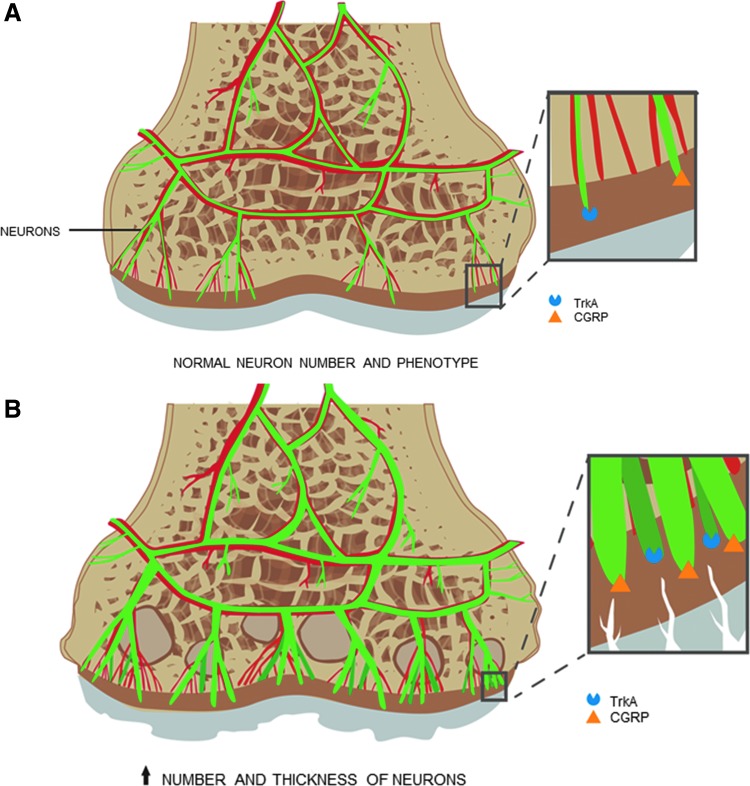
Innervation of the osteochondral unit. In synovial joints, sensory neurons innervate the healthy subchondral bone and show a normal expression of TrkA and CGRP **(A)**, which are important effectors in the transmission of nociception. In osteoarthritic joints, neurons increase in number and size, and TrkA and CGRP are upregulated **(B)**. TrkA, tropomyosin receptor kinase A; CGRP, calcitonin gene-related peptide.

Nerve growth was further confirmed in a rat model, where upregulation of calcitonin gene-related peptide and tyrosine receptor kinase A (both nociceptive markers) concurrent with increased size of neurons was found in the subchondral bone of OA knee joints (Fig. 3).^35^ NGF and its effects are important mediators of pain,^36^ thus increases in NGF and nerve growth associated with angiogenesis in osteochondral tissue are likely causes of joint pain.

It remains unclear at what point subchondral bone damage and/or focal lesions present as pain, making it difficult to diagnose and treat before further tissue degeneration and OA progression. However, the presence of asymptomatic bone marrow lesions can predict cartilage loss and subsequent OA symptoms.^37^ Cartilage loss leads to subchondral bone exposure within the osteochondral unit, which may also be associated with pain. Moisio *et al.* conducted cross-sectional analyses of patients with OA to determine the association of subchondral bone exposure (as evidenced by MRI) with knee pain, and found that moderate-to-severe knee pain was associated with the percentage of denuded bone, suggesting a relationship between the extent of subchondral bone exposure and increased pain.^38^

These results suggest that exposure of the bone can result in contact with the synovial fluid or rubbing with other structural components of the joint, leading to biochemical and/or mechanical stimulation of subchondral bone nociceptors, which may partly explain the pain associated with damage to the osteochondral unit. Lastly, subchondral bone sclerosis may contribute to increased vascular pressure in the marrow cavity and subchondral bone plate. This is exacerbated by inactivity, and may be a contributing factor for night pain in OA.^39,40^ All of this evidence points to the important role of the subchondral bone in the progression of joint pain and cartilage damage, making it a crucial therapeutic target for both pain relief and structural restoration of the osteochondral unit.

### Evaluation of the osteochondral unit

Once patients are experiencing symptoms of joint pain and limited mobility, evaluation of the affected joint is necessary to determine the extent of damage (if any) to the osteochondral unit and its associated tissues. Generally, radiography with the patient weightbearing is the most commonly used initial imaging technique for the diagnosis of OA.^41^ OA severity is often graded using the Kellgren and Lawrence system, which scores the presence and severity of osteophytes, joint space narrowing, sclerosis, and other bone changes.^42^

However, radiography cannot detect early pathological bony changes and does not depict soft tissues such as cartilage. Radiographs therefore do not provide a complete evaluation of osteochondral lesions preceding or accompanying OA, and are not a sufficient imaging modality to determine the appropriate treatment of the damaged osteochondral unit.

The gold standard for noninvasive imaging of the joint is MRI. MRI can supply a whole organ assessment of both hard and soft tissues in the joint, and it eliminates patient exposure to radiation. Many studies have determined that high-field MRI (with a greater signal-to-noise ratio) provides a more sensitive and reliable image that can more easily detect small, early stage lesions in the chondral phase.^43–46^

To further quantify cartilage composition, advanced MRI techniques, such as T2 mapping can be used. T2 mapping is a relaxometry measurement that reflects the water content, collagen content, and fiber orientation in articular cartilage. More specifically, it can be used to detect early tissue degeneration, as increases in T2 relaxation times have been correlated with improper collagen stratification.^47^ While still mostly only utilized in a research setting, T2 mapping has shown promising results in predicting the development of macroscopic changes visible with conventional MRI. One prospective study found that T2 mapping significantly improved detection of cartilage lesions over routine MRI, indicating its potential for use in diagnosing and monitoring early cartilage degeneration.^48^

Arthroscopy has an advantage over other imaging techniques in that it allows for direct observation of the pathology of the affected joint. However, it does not allow assessment beyond the cartilage surface layer, except for determining tissue integrity. Therefore, this minimally invasive procedure is usually performed if the MRI results on soft tissue are inconclusive,^49^ as it can be difficult to evaluate early cartilage damage on MRI because of the tissue's thinness, even with advances such as T2 mapping. For the diagnosis of osteochondral lesions of the talus, MRI and arthroscopic evaluation results were well correlated,^50,51^ however, the diagnosis for large chondral defects should be confirmed with arthroscopy, as MRI alone can provide false-positive or false-negative findings.^52,53^

Arthroscopy can also serve as a one-time surgery for both diagnosis and treatment, and thus is indicated when noninvasive imaging techniques suggest that surgical intervention may be necessary to debride or repair cartilage. It is important to consider that there may be incomplete or no correlation between clinical symptoms, such as pain and diagnostic imaging,^2^ which complicates the therapeutic approach taken when aiming to alleviate symptoms and/or repair an asymptomatic lesion. An invasive treatment may actually worsen pain.^54^

### Surgical treatment of osteochondral unit damage/disease

At present, there are no pharmaceutical drugs to treat chondral, subchondral, or osteochondral lesions. In the case of focal osteochondral injuries or severe joint disease, surgical intervention is the only treatment option for structural restoration and possible reduction of pain. Surgical restoration is an extremely complex process, largely due to the unique structure, mechanical strength, and crosstalk within the osteochondral unit and also with adjacent tissues.

Currently, microfracture, ACI, or variations of ACI, such as matrix-associated or gel-associated chondrocyte implantation are used to treat focal chondral lesions. In humans, microfracture has shown success in improving pain and function outcomes in the majority of patients treated, although the majority of the repair tissue was fibrocartilaginous.^55^ However, upon short- (1 year) and long-term (4–6 years) follow-up, patients treated with microfracture for talar osteochondral lesions showed deteriorating subchondral bone health, with the development of cysts and subchondral bone plate thickening long term.^56,57^

Even though the technique of ACI does not involve direct intervention into the bone, it has also been associated with complications in the subchondral bone. Subchondral bone plate advancement, intralesional osteophytes, and subchondral bone cysts were found in 30–60% of human patients treated with ACI after mid- to long-term follow-up.^58–60^ The upward migration of the subchondral bone due to the renewal of endochondral ossification at the tidemark has been shown to be associated with the degradation of the repaired articular surface.^61^

Particulate, chip, or minced tissue has been explored for cartilage resurfacing and repair of the osteochondral unit in a number of studies. For example, BioCartilage™ is a relatively new procedure, whereby minced cadaveric allogeneic cartilage (sourced from adult donors) is implanted into a microfracture site along with platelet-rich plasma for its perceived anti-inflammatory and anticatabolic effects.^62^ Early results in horses suggest that BioCartilage-treated full-thickness defects have greater collagen type II content and better repair/host integration than traditional microfracture, although both groups had subchondral bone voids at the defect site.^63^

A similar technique, DeNovo^®^ NT, involves implanting particulate cartilage from juvenile donors (typically younger than 2 years) delivered in fibrin glue. This method relies on the observation that juvenile chondrocytes induce matrix formation in adult cartilage.^64^ Although subjectively assessed, this allogeneic technique has shown good clinical outcomes in the treatment of osteochondral lesions in the knee^65^ and ankle^66^ in small retrospective studies. However, MRI and histological results demonstrated an uneven cartilage surface, subchondral bone edema, and heterogeneous repair tissue despite improved patient scores. A recent study comparing DeNovo NT and microfracture in talar osteochondral defects found no differences in patient-reported outcomes or repair tissue quality.^67^

A new autologous strategy has shown some success as well. CAIS involves isolating cartilage tissue from a healthy nonweight-bearing area, mincing the cartilage, then implanting it into the defect site in a one-step procedure. CAIS was compared with ACI in horses to treat large defects (15 mm in diameter). Both techniques resulted in superior tissue regeneration compared with empty defects, with CAIS having the higher score.^68^ In humans, CAIS outperformed microfracture in a randomized controlled trial in knee function, reduced pain and stiffness, sports and recreational activities, and knee-related quality of life.^69^

In addition to morselizing autologous or allogeneic cartilage, a recent report has described the efficacy of implanting both fragmented bone and cartilage into osteochondral defects. Christensen *et al.*^70^ utilized autologous bone fragments press-fitted into defect beds with cartilage chips embedded in fibrin glue seeded on top. After 12 months, all eight patients had improved Magnetic Resonance Observation of Cartilage Repair scores in defect fill and cartilage tissue surface, as well as good patient-reported outcomes, although subchondral bone-specific scores (edema, bone interface) were not significantly improved.^70^

The authors further elucidated the role of the cartilage chips in the quality of the repair tissue. In a minipig model, defects treated with autologous bone fragments and cartilage chips had significantly more hyaline and fibrocartilage than defects treated with bone fragments alone, although no difference was noted in the bone defect volume between the groups at 6 or 12 months.^71^

OAT and mosaic arthroplasty have a distinct advantage over the aforementioned techniques in that they aim to repair both the cartilage and the bone phase with healthy, intact osteochondral grafts. Both techniques involve harvesting one (OAT) or more (mosaic arthroplasty/mosaicplasty) osteochondral plugs from a healthy, nonweight-bearing area of the joint and implanting it into the lesion. OAT is often indicated when a medium (∼2–3 cm^2^) full-thickness defect or multiple lesions are diagnosed through MRI or arthroscopy.^72,73^

This technique was pioneered in horses by Bodo *et al.* to treat subchondral bone cysts, and has shown promise in the treatment of focal cartilage defects in dogs, pigs, and sheep.^73–77^ In a recent systematic review, 72% of human patients who underwent OAT in the knee joint (10 studies, 610 patients) had successful clinical outcomes after an average of 10 years (based on Lysholm/International Knee Documentation Committee [IKDC] scoring), although activity scores did not improve significantly from preoperative to final follow-up.^78^ While the average return-to-sport rate was reported to be 85% in the studies that assessed this, highly active patients reported lowering their activity level, while sedentary patients did not change their activity level.^78,79^ Mean failure rate among the studies reviewed was 28%. Failure rates are influenced by age, lesion size, and/or localization, and concomitant surgeries,^80^ which also act as compounding variables when evaluating OAT as an effective treatment protocol for OCLs.

To date, seven randomized studies (evidence level: I or II) have compared OAT with microfracture in pediatric and adult patients.^81–87^ Out of these studies, five demonstrated that OAT produced better clinical outcomes and a higher return-to-sport rate than microfracture at mid- (3–5 years) to long-term (≥10 years) follow-up.^81–84,87^ Two studies did not find significant differences in clinical outcomes between OAT and microfracture at mid- (5 years) and long-term (10 years) follow-up, however, they were both level II randomized studies with a small number of patients in each group.^85,86^

There have been even fewer studies published on OAT versus ACI. To the best of our knowledge, only four randomized trials have been conducted to date, with mixed outcomes.^85,88–90^ After 1 year, Bentley *et al.* found that ACI-treated patients had better clinical results than mosaicplasty-treated patients,^88^ while Horas *et al.* determined that OAT was superior to ACI, delivering higher Lysholm scores after a 2-year follow-up.^89^ Dozin *et al.* could not conclude any significant differences between mosaicplasty and ACI, although they reported a higher Lysholm score for mosaicplasty patients after 2 years (88% vs. 68% for ACI patients).^90^ Lim *et al.* compared OAT versus ACI versus microfracture in 70 knees, and did not observe any significant differences in clinical outcomes between the three treatments after 5 years.^85^ To date, there have been no published Level I clinical trials comparing the long-term outcomes of OAT versus ACI.

Major concerns with OAT with respect to donor-site morbidity and critical lesion size can be addressed by using allografts harvested by cadaver limbs. Osteochondral allografts have increased in popularity as long-term storage protocols have been optimized and immune rejection rates remain low.^91,92^ A single-center, randomized, prospective trial comparing the efficiency of autografts versus allografts to heal large and/or recurrent osteochondral lesions of the talus found similar healing rates.^93^ However, as for all human donor-sourced material, adequate supply of allografts is a concern for widespread adaptation and rollout.

### Osteochondral unit tissue engineering

Sourcing appropriate healthy allograft tissue remains a key limitation to osteochondral allograft transfer.^94^ Fortunately, much progress has been made in the realm of tissue engineering of osteochondral constructs. The majority of these constructs contain cells and one or more types of scaffolds for the cartilage and/or bone phase. Various studies have investigated methods for generating biphasic, multiphasic, and/or gradient tissues (reviewed in refs.^95–99^), different biomaterials/scaffolds (reviewed in refs.^100,101^), as well as the controlled release of growth factors for *in vivo* tissue regeneration (reviewed in refs.^102,103^). While these *in vitro* studies show promise, relatively few *in vivo* studies have been conducted to show how engineered osteochondral constructs function long-term in the joint.^104–109^

Questions remain concerning methods to reproducibly generate stable, mechanically competent, metabolically equivalent, and functionally relevant osteochondral units. Generally speaking, most publications report that the compressive and/or shear modulus of tissue-engineered cartilage is inferior to that of native cartilage. While biomechanical competency of cartilage is important in the creation of implant-grade tissue, the integration to and vascularization of the new subchondral bone is imperative in recapitulating the health and function of the native osteochondral unit. By focusing on building the unit as a whole, we can eventually overcome some of the limitations observed by attempting to regenerate only the chondral phase.

### Animal models, human trials, and the case for health technology value in osteochondral unit repair

Research into pathologies and treatment of human cartilage disease conditions often utilizes animal models as a transition phase between *in vitro* studies and human clinical practice. Small animal models such as mice and rats are most commonly used as they are inexpensive and can be generated as transgenic or gene knockout animals, which allow for the exploration of direct or indirect genetic causes of cartilage-associated pathologies.^110^ However, while they are suitable models for hypothesis-generating studies of OA pathology and development, their small joint size and relatively thin articular cartilage means it is difficult to study clinically relevant-sized defects. Larger animals, such as rabbits, goats, pigs, sheep, dogs, and horses have also been studied extensively as models for cartilage repair (reviewed in Chu *et al.*^111^).

When choosing a large animal model for the treatment of osteochondral defects, several factors must be taken into consideration. Joint anatomy, cartilage thickness, subchondral bone properties, and biomechanical loading environment should be as close as possible to humans. Goat and sheep stifles are very similar in anatomy to the human knee, however some differences (mainly the femoral intercondylar notch width as well as trochlear groove length) limit very precise modeling of the human joint.^112^ In terms of cartilage thickness, horses are most similar to humans with a thickness of 1.75–2 mm^113^; human cartilage thickness ranges from 2.4 to 2.6 mm.^114^ Unlike normal human subchondral bone, horses and goats have higher bone mineral density, bone volume fraction, and thicker trabeculae.^115^

However, as these are features of OA,^116^ they may in fact pose an advantage to developing osteochondral defect treatments, where the subchondral bone environment is altered. Goats have similar joint biomechanics to humans, whereas horse joints are subjected to increased loads as a result of the animal's size and activity level,^117^ making defect repair particularly challenging yet perhaps excellent for modeling repair.

In addition to their similar joint characteristics to humans, horses have been deemed the superior preclinical animal model due to analog developmental disorders of the osteochondral unit and their propensity to acquire joint injuries through athletic activities.^118,119^ In addition, arthroscopic techniques to treat and evaluate lesions in the horse are similar to human surgical instrumentation, aiding in technology transfer between horses and humans.^120^ Horses are also amenable to controlled rehabilitation protocols, an integral postoperative part of many surgical treatment approaches.

Veterinary patients also suffer from natural spontaneous disease, which has significant value in preclinical modeling for assessing the true clinical importance of new therapies. Assessing new cartilage and osteochondral defect treatments in these patients is beneficial because they can be compared with current best clinical practices. In addition, disease conditions are associated with all of the complexities of human patients, such as chronicity, comorbidities, polypharmaceutical use, epigenetic factors, genetic heterogeneity, and postsurgical compliance issues, as reviewed elsewhere.^121^

Recently, it has been highlighted that human research studies and clinical trials are often conducted in younger people, whereas the majority of patients for these procedures are older.^122^ Only 4% of patients in a clinical practice fulfill inclusion criteria of clinical trials on focal cartilage repair.^123^ This likely reflects bias toward early commercial or research successes, but is ultimately failing development of effective treatments.^122^

Of note, some now argue that cartilage repair should be exempt from the rigor of randomized clinical trials (RCTs).^124^ Complete replacement of all animal studies with microdosing strategies in humans has also been advocated recently.^125^ However, this argument seems more relevant to traditional drug testing than cell and tissue-engineering strategies.

Instead of abandoning the rigor of RCTs and arguing for regulatory lenience in approving new cartilage repair methods, better strategies to determine safety and efficacy of new treatments in a cost-effective manner should be explored. In addition to enhancing the welfare of our veterinary patients, such an approach may also allow for separate veterinary revenue streams that could be used to support clinical trials in humans.

## Conclusion

When pathologies arise in one component of the osteochondral unit, reducing its function, it is often only a matter of time before the other components are affected and OA occurs. Early detection and diagnosis of osteochondral lesions is important for the appropriate treatment of the disease. Unfortunately, current nonsurgical treatments are only capable of temporarily relieving symptoms and perhaps slowing down joint disease progression, whereas surgical treatments come with a plethora of complex difficulties, limitations, risks, and expenses. By using treatment techniques that focus solely on repairing the cartilage surface and do not address existing or future changes to the subchondral bone, pain can persist and cartilage damage may reoccur. The possible result is a joint that looks normal when observed arthroscopically, yet the patient still complains of joint pain.^32^

Further development of diagnostic tools, disease-modifying drugs, and surgical alternatives is necessary for a more effective, targeted therapeutic approach that can reverse disease progression and restore joint function long term, without the need for life-long treatment or revision surgeries. Basic research into the complexities of cartilage/bone crosstalk and the *in vivo* development of the osteochondral unit is fundamental to the improvement of joint therapies and tissue restoration. From a clinical and translational perspective, naturally occurring disease in companion animals, such as horses and dogs, provides unique opportunities for developing and assessing the long-term safety and efficacy of new osteochondral treatments to the benefit of animals themselves as well as humans. Rarely is osteochondral pathology a fatal condition and new therapies should therefore be thoroughly evaluated for safety and efficacy before implementation in human medicine.
